# Fiber Melt Spinning and Thermo-Stabilization of Para-Rubber Wood Lignin: An Approach for Fully Biomass Precursor Preparation

**DOI:** 10.1021/acsomega.3c04590

**Published:** 2023-09-06

**Authors:** Prapudsorn Wannid, Bongkot Hararak, Sirada Padee, Wattana Klinsukhon, Natthaphop Suwannamek, Marisa Raita, Verawat Champreda, Chureerat Prahsarn

**Affiliations:** † National Metal and Materials Technology Center (MTEC), National Science and Technology Development Agency (NSTDA), 114 Paholyothin Road, Klong Luang, Pathum Thani 12120, Thailand; ‡ National Center for Genetic Engineering and Biotechnology (BIOTEC), National Science and Technology Development Agency (NSTDA), 113 Paholyothin Road, Klong Luang, Pathum Thani 12120, Thailand

## Abstract

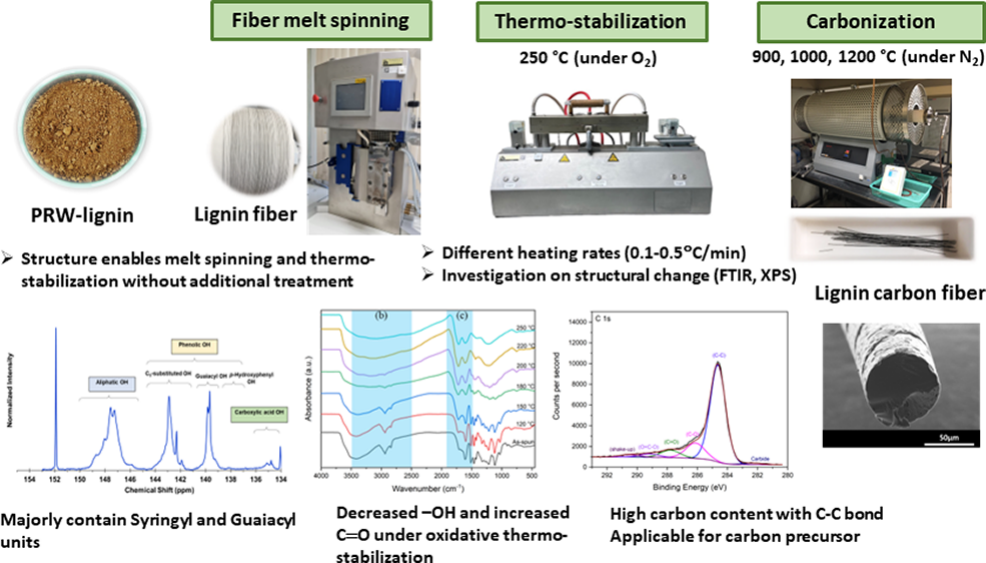

Para-rubber wood (PRW) lignin, extracted from agricultural waste, was successfully melt-spun to fibers and thermo-stabilized without employing auxiliary additives. ^31^P NMR analysis revealed that PRW-lignin contained mainly a syringyl unit of phenolic C_5_-substituted OH group, which enabled melt flow during fiber spinning, as well as a guaiacyl unit which offered the ability to cross-link during thermo-stabilization. Thermo-stabilized fibers with no fusion were achieved at 250 °C with the heating rate of 0.1 °C/min. Structural changes in the fibers during stabilization were systematically investigated using FTIR and XPS analyses. From the results, changes in the intensities of characteristic bands relating to C–H stretching, aromatic C–H stretching, and C=O stretching indicated structural changes of lignin toward aromaticity via oxidation reactions. XPS analysis of the fibers carbonized at 900, 1000, and 1200 °C revealed an increase in carbon content from 72 to 87 wt %. and a decrease in oxygen content from 28 to 13 wt %. with the increasing carbonization temperature. The weight loss of carbonized fibers was in the range of 73.6 to 88.7%. The high weight loss of fibers carbonized at 1200 °C was explained partly due to the thermal decomposition of disordered carbon. The tensile strength and modulus of carbonized fibers were 163.0 and 275.1 MPa, respectively. This study demonstrates an approach to prepare a fully biomass precursor fiber and contributes to the exploration of the potential use of lignin from biomass waste.

## Introduction

Lignin is a complex heteropolymer, composed of amorphous polyphenolic macromolecules with varied functional groups.^1−10^ Its heterogeneous aromatic structures comprised mainly of three phenyl propanoid units: *p*-hydroxyphenyl (H), guaiacyl (G), and syringyl (S) units, linked by various carbon–oxygen and carbon–carbon bonds such as β–O–4, 5–5, 4–O–5, and β–β, to form three-dimensional structures.^1−13^ Variation and complexity in the molecular structures and compositions of lignin are derived not only from inherited factors such as plant types and plantation regions but also external factors such as extraction and recovery processes.^1,2,5−10^ This has been a great challenge for lignin conversion and processing for desired applications.

Among various potential applications, lignin has gained considerable attention as an alternative bioderived precursor for carbon fiber production due to its abundance in nature, low cost, renewability, and high carbon content.^1,6,10−15^ To prepare lignin fibers, several techniques including melt spinning, dry spinning, wet spinning, and electrospinning could be employed. In terms of production, melt spinning of lignin fibers has been highly attractive due to its advantage on the economy of scale and solvent-free process.^11,13,14^ In melt spinning, the melted lignin is extruded through the spinneret’s holes and drawn to fibers. It is crucial that a proper processing temperature, beyond glass-transition temperature (*T*_g_), should be employed to melt lignin without significant depolymerization and/or condensation, which otherwise could cause defects on fiber surfaces and/or poor fiber spinnability.^16^ The obtained lignin fibers must possess the ability to cross-link during the thermo-stabilization step so that the fibers can survive the ultimate high temperature in the carbonization step. Different lignins possess different molecular structures and properties and thus behave differently during melt spinning and conversion to carbon fibers.^5,13,17^ Understanding the molecular structures as well as characteristics under the processing of lignin is essential to unleash its potential applications.

Research works on the preparation of lignin fiber precursors via melt spinning revealed that the structural and thermal properties of lignins greatly affected their melt spinning and thermo-stabilization processes. The obtained fibers exhibited different features and properties, depending on the lignin characteristics as well as the processing parameters employed. Baker and Rials^14^ prepared carbon fiber precursors from kraft hardwood lignin (HWL) and organic-purified hardwood lignin (HWL-OP) via melt spinning. Results showed that HWL-OP exhibited better fiber spinnability than HWL due to its lower *T*_g_ (86 °C vs 133 °C) and the larger proportion of low-molecular-weight fraction. The obtained lignin fibers were thermo-stabilized at 250 °C, using a very low heating rate (0.01 °C/min), followed by carbonization at 1000 °C. The obtained carbon fibers exhibited a tensile strength of 0.517 GPa. In continuing work, HWL with a narrow molecular weight distribution was thermally pretreated to improve its *T*_g_ and melt flow. The tensile strength of the obtained carbon fibers could be increased to 1.07 GPa. Kubo et al.^18^ reported that hardwood acetosolv lignin, with *M*_w_ of 4800 g/mol, PDI of 2.7, and glass-transition temperature (*T*_g_) of 128 °C, could be melt-spun into fibers with an average diameter of 24–28 μm. During thermo-stabilization, fiber fusion was observed in the hardwood acetosolv lignin. On the other hand, softwood acetosolv lignin with a lower molecular weight (4400 g/mol) and narrower PDI (2.4) was prepared by treating with aqueous acetic acid. Its *T*_g_ was lower than that of pristine lignin. This enabled the melt spinning of softwood lignin into fibers. The obtained precursor fibers could be converted to carbon fibers without fiber fusion. Similar work was also reported on preparing lignin with a lower molecular weight, narrow PDI, and lower *T*_g_, using other organic solvents such as acetone,^19,20^ methanol,^21^ and ethanol.^22^

Improvement in the melt flow properties of infusible lignins had been done by adding thermoplastic polymers such as polypropylene,^23,24^ polyethylene,^25,26^ polyester,^24^ polyvinyl alcohol,^27^ polyethylene oxide,^28^ and poly(lactic acid).^29,30^ The amount of carrier polymers added varied from 3–5 wt % up to 30 wt %. Kadla et al.^23^ reported the preparation of carbon fibers from three lignins: organosolv lignin (Alcell), softwood kraft lignin (SWKL), and hardwood kraft lignin (HWKL). Different amounts of PEO (5, 12.5, and 25 wt %) were blended with lignins to improve the fiber spinnability. In melt spinning, SWKL did not melt and tended to cross-link during heating and thus could not be extruded to form fibers. Thermo-stabilization at 250 °C revealed that HWKL and HWKL/PEO fibers were more thermo-stable than Alcell and Alcell/PEO. HWKL/PEO fibers, containing PEO less than 5 wt %, could be thermo-stabilized without fiber fusion. After carbonization, the obtained carbon fibers exhibited tensile strength in the range of 0.388–0.458 GPa. Thunga et al.^29^ modified the softwood kraft lignin by butyration before blending with 0, 10, 25, and 50 wt %. poly(lactic acid) and melt spinning into fibers. The lignin fiber precursors were converted to carbon fibers by thermo-stabilization at 250 °C, followed by carbonization at 1000 °C. Porous morphologies caused by PLA depolymerization during carbonization were observed inside the fibers. Void defects observed in carbon fibers were also reported by Wang et al.^31^ In addition, Luo et al.^32^ demonstrated a new approach to improve the molecular orientation of lignin during fiber melt spinning via the preparation of lignin-based acrylate polymer from red oak lignin. The obtained carbon fibers exhibited a tensile strength of 1.70 GPa and modulus of 182 GPa.

The great challenges for the preparation of melt-spun lignin fiber precursor were majorly contributed from the opposite characteristics required to achieve simultaneously.^16^ Lignin should have low enough *T*_g_ to be melt-spun, yet high enough *T*_g_ to be thermo-stabilized rapidly. Furthermore, its chemical structure should be stable enough so that it can be melt-spun at elevated temperatures yet active enough to cross-link thoroughly during thermo-stabilization. In the fiber precursor preparation, thermo-stabilization is considered the most critical step, where the fibers must undergo cross-linking with increasing temperature without fusing to one another. Incomplete cross-linking as well as surface fusion can lead to defects on the prepared carbon fibers, which can reduce their strength drastically.

The processability and final properties of lignin fibers are largely affected by the chemical structure, molecular weight, PDI, and impurity of lignin, as well as processing conditions. To improve the spinnability of lignin and the properties of lignin fibers, it is crucial to understand the relationships among lignin structures, spinning parameters, and properties of the fibers. In our previous work,^33^ chemical structures, characteristics, and properties of lignins from three Thai biomass including bagasse (BG), palm kernel shell (PKS), and para-rubber wood (PRW) were investigated in order to process them effectively. From the results, it was observed that lignin from para-rubber wood (PRW-lignin) exhibited good melt flow during fiber spinning, as well as the ability to cross-link upon heating during the thermo-stabilization process. This enabled the preparation of the lignin fiber precursor where no auxiliary additive was needed.

In this continued work, preparation of a fully biomass fiber precursor from PRW-lignin was explored. ^31^P NMR and DSC were employed to confirm the chemical structures, compositions, and glass-transition temperature (*T*_g_) of lignin. The PRW-lignin fibers were prepared via melt spinning. Thermo-stabilization and carbonization of the melt-spun PRW-lignin fibers were conducted, and changes in their chemical structures were also characterized. Insight knowledge about the preparation of Thai biomass lignin fibers could unleash the potential of biomass utilization and contribute to global green economy.

## Experimental Methods

### Materials and Chemicals

PRW-lignin was obtained via organosolv fractionation of agricultural residues. PRW was supplied by Asia Biomass Public Co., Ltd. The preparation of PRW-lignin is described in detail elsewhere.^33,34^ In brief, PRW residues were milled and sieved to 2–4 mm in size before being dried at 70 °C overnight. The mixed solvent of ethanol and water (70:30%v/v) with 1%w/w sulfuric acid (H_2_SO_4_) was employed for lignin fractionation. The process was carried out at 175 °C under N_2_ at a pressure of 20 bar. After 60 min, the reaction was stopped by quenching on ice for 15 min, and the slurry was sieved to separate the solid fractions. The recovered organosolv lignin was then filtered and dried at room temperature for 3 days. It was worth noting that PRW-lignin employed in this work was produced in a different lot from that employed in our previous reported work. The measured physical and thermal properties, therefore, were slightly different.

### Melt Spinning of PRW-Lignin

Fiber melt spinning of PRW-lignin was carried out using a microcompounder (MC 40, Xplore). Prior to spinning, lignin was dried at 80 °C in a vacuum oven for 12 h to remove moisture. Based on the thermal characteristics previously reported,^33^ fiber melt spinning of PRW-lignin was conducted at a processing temperature of 165 °C, under N_2_ flow (1 L/min). The melted lignin was extruded, using a screw speed of 5 rpm, through a spinneret with a hole diameter of 0.5 mm and continuously collected at a winding speed of 10 m/min.

### Thermo-Stabilization and Carbonization of PRW-Lignin Fibers

Oxidative thermo-stabilization of the melt-spun PRW-lignin fibers was conducted, using a lignin fiber line conditioning unit (Xplore, Lignin fiber line, The Netherlands). Lignin fibers were heated to 250 °C at different heating rates (0.1, 0.2, 0.3, 0.4, and 0.5 °C/min) under O_2_ (flow rate of 1 L/min) and held for 30 min at 250 °C. Changes in the chemical structures and thermal properties of the fibers during thermo-stabilization were investigated by heating lignin fibers to 250 °C, using a heating rate of 0.1 °C/min, and sampling out the fibers after the stabilization temperature reached the determined values (120, 150, 180, 200, 220, and 250 °C) for FTIR and DSC analyses.

Carbonization was conducted at three different temperatures (900, 1000, and 1200 °C), using a tube furnace (Protherm tube furnace, 1600 °C, Turkey). The thermo-stabilized PRW-lignin fibers were heated from 30 to 150 °C at a heating rate of 2 °C/min under N_2_ (flow rate of 2 L/min) and kept at 150 °C for 1 h to remove the moisture residual. Temperature was then ramped up to the desired carbonization temperature (900, 1000, and 1200 °C) at a heating rate of 10 °C/min and kept isothermally for 1 h. The carbonized fibers were then cooled down under N_2_ atmosphere until the temperature reached 400 °C. The N_2_ gas was then turned off to allow cooling to room temperature under ambient air.

### Characterizations

Lignin content was determined according to the laboratory analytical procedure provided by the National Renewable Energy Laboratory (NREL).^35^ Determination of ash in PRW-lignin was conducted using the laboratory analytical procedure, NREL/TP-510-42622.

The molecular structure and compositions of PRW-lignin were investigated using ^31^P NMR (AV-500 Bruker Biospin). The hydroxyl groups in PRW-lignin were converted to phosphitylated products, of which the protocol of phosphitylated lignin solution preparation followed the steps reported in ref (36). Mixture solvent A was prepared by mixing anhydrous pyridine/deuterated chloroform in the ratio of 1.6:1 v/v. 5 mg of chromium(III) acetylacetonate and approximately 18 mg of endo-N-hydroxy-5-norbornene-2,3-dicarboximide (NHND) were loaded into 1.0 mL of the as-prepared solvent A. The actual weight of NHND was recorded, and the resulting solution was referred to as an internal standard (IS) solution. The IS solution (0.1 mL) was placed into an air-tight glass vial with a PTFE-lined septum, and the actual weight of the IS was recorded. For sample preparation, 30 mg of predried PRW-lignin and 0.5 mL of solvent A were then added to the aforementioned vial with constant stirring for 12 h. After complete dissolution, 0.1 mL of 2-chloro-4,4,5,5-tetramethyl-1,3,2-dioxaphospholane (TMDP) was added, and the vial was sealed with moisture-resistant Parafilm. The vial was then shaken using a vortex mixer II G560E shaker for 2 min to ensure thorough mixing. The obtained phosphitylated lignin solution was transferred into a 5 mm NMR tube for subsequent ^31^P NMR characterization. The acquisitions consisted of 256 scans with a 5 s relaxation delay time. The resulting spectra were analyzed using Bruker Topspin software.

The determination of molecular weight and molecular weight distribution of PRW-lignin was conducted by GPC (Waters e2695, Waters Corporation, USA). In testing, a sample solution of 10 mg lignin in 5 mL of tetrahydrofuran was prepared and filtered through a 0.2 pore-sized membrane before being injected into GPC columns (flow rate of 1 mL/min, 25 °C). The weight-averaged and number-averaged molecular weight (*M*_w_ and *M*_n_) as well as polydispersity index (PDI) were obtained.

The appearance of the attained fibers was evaluated by a field emission scanning electron microscope (model SU5000, Hitachi, Japan), using an acceleration voltage of 5 kV. The fibers were gold-coated for 60 s by Quorum-Q150RS (UK). Elemental composition (wt %.) of the fibers was determined by energy-dispersive X-ray analysis (EDX, X-Max, Horiba, Japan) at an acceleration voltage of 15 kV, with a BSE detector and a working distance of 10.7 mm. Data evaluation was done with Esprit v.1.9.3. software (Bruker Corp., USA).

Fourier transform infrared spectroscopy (FTIR, Spectrum Spotlight 300, Perkin Elmer, USA) was employed to identify and analyze the changes in functional groups in PRW-lignin fibers during thermo-stabilization. The lignin fibers were sampled out after the stabilization temperature reached the determined values (120, 150, 180, 200, 220, and 250 °C) for FTIR investigation. In sample preparation, 1.5 mg of lignin fiber was mixed with 100 mg of potassium bromide (KBr) and ground together into a fine powder before being transferred into a compression die, which was then pressed under a high pressure to form a sample disc. FTIR characterization was conducted in transmission mode, following ASTM (E1252–98). The spectra were obtained using an average of 16 scans at a resolution of 4 cm^–1^ in the wavelength range of 4000 to 400 cm^–1^.

Glass-transition temperatures (*T*_g_) of melt-spun PRW-lignin and stabilized PRW-lignin fibers were investigated using a differential scanning calorimeter (Mettler Toledo: DSC1, Switzerland). In testing, the lignin sample (5–10 mg) was placed in an aluminum pan and heated up and cooled down (at the rate of 10 °C/min) under N_2_ flow (20 mL/min) for two cycles in the following conditions. The first cycle: 40–105 °C, kept for 5 min, cooled to 40 °C, and kept for 1 min. The second cycle: 40–250 °C, kept for 1 min, cooled to 40 °C, and kept for 1 min. The *T*_g_ values of lignin samples were determined from the thermograms of the second cycle.

XPS was conducted using a Kratos Axis Supra instrument (Shimazu Group Company, Kratos Analytical Ltd., UK) equipped with an Al Kα X-ray source (*h*ν = 1486.6 eV) monochromator. The analysis of XPS spectra was done to investigate the changes in the chemical structure of lignin fibers during thermo-stabilization and carbonization processes. For XPS spectra examination, the energy employed was 160 eV for a wide survey scan and 40 eV for a narrow or high-resolution scan of the C 1s and O 1s spectra. Spectral deconvolution was performed using the CasaXPS program. The C 1s spectrum was deconvoluted employing a Shirley-type background, and curve-fitting was carried out using a combined Gaussian/Lorentzian function (70/30 ratio). The full width at half-maximum (FWHM) of the deconvoluted components was determined. Specifically, a binding energy of 285.0 eV was attributed to the C–C bond within the C 1s spectrum.

Raman analysis was performed using a dispersive Raman microscope (Bruker Optics, Senterra) equipped with a laser excitation wavelength of 532 nm, a power laser at 2 mW, spectral range of 4500–70 cm^–1^, and a TE-cooled CCD detector.

Tensile testing of the thermo-stabilized and carbonized PRW-lignin fibers was attempted to explore their mechanical properties, using an Instron universal testing machine (5943, USA). In testing, single fibers were set up at a gauge length of 25 mm and pulled at a speed of 2 mm/min with a 10 cN load cell. The averaged tensile strength and Young’s modulus were reported.

## Results and Discussion

The molecular structure, composition, as well as thermal properties of PRW-lignin were characterized to understand their influence on the melt spinning process. The PRW-lignin fibers were then prepared via melt spinning. These were described as fully biomass fiber precursors because PRW-lignin could be processed in melt spinning and thermo-stabilization without the addition of carrier polymers/cross-linking agents or additional treatment such as thermal pretreatment. Thermo-stabilization and carbonization of the PRW-lignin fiber were conducted to explore its utilization as a carbon fiber precursor.

**Figure 1:**
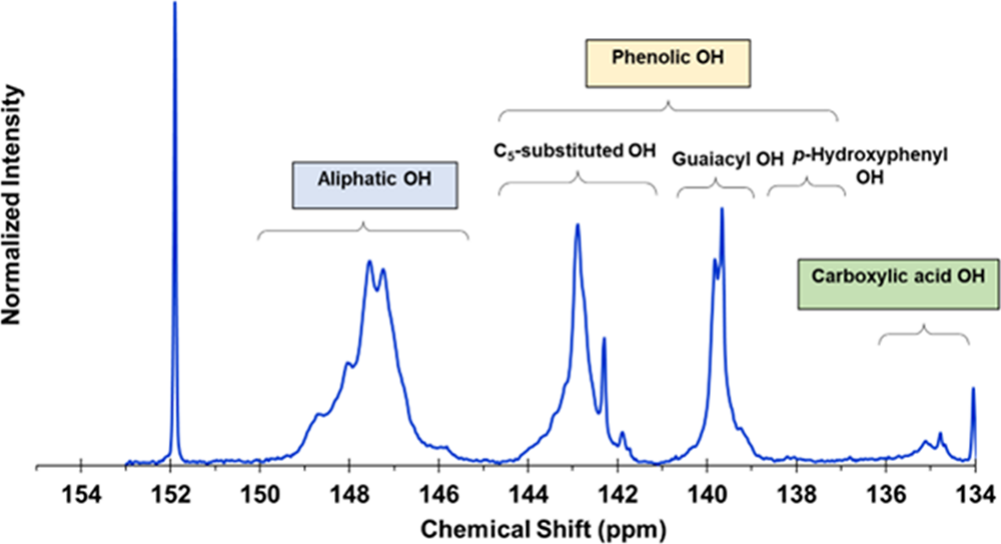
^31^P NMR spectrum of PRW-lignin with signal assignments.

The determined lignin content and ash content of PRW-lignin were 98.86 and 0.22%, respectively (Klason method). ^31^P NMR spectroscopy was performed to quantitatively analyze the phenolic and nonphenolic (aliphatic) structures as well as different forms of phenolic units (H, G, S) in PRW-lignin. The assessment of distinct phenolic units was achieved via the determination of phosphitylated hydroxyl groups.^37,38^ The hydroxyl groups in lignin reacted with the phosphitylating agent (2-chloro-4,4,5,5-tetramethyl-1,3,2-dioxaphospholane, TMDP) in the presence of an organic base (pyridine), resulting in the formation of phosphitylated hydroxyl groups. The ^31^P NMR spectrum of the PRW-lignin sample with the assigned main signals is shown in Figure 1. Three main features of aliphatic, phenolic, and carboxylic hydroxyls defined at the chemical shifts of 145.4–150.0, 137.6–144.0, and 133.6–136.0 ppm, respectively, were observed. Two chemical shifts of phenolic units, corresponding to C_5_-substituted and guaiacyl units, were detected at 140.0–144.5 ppm and 139.4 ppm, respectively. Quantitative analysis of lignin diversity was achieved by integrating the signal area, utilizing N-hydroxy-5-norbornene-2,3-dicarboximide as the internal standard.^37,39^ From the results, it is observed that PRW-lignin contained an aliphatic OH group (2.15 mmol g^–1^), a phenolic OH group (1.59 mmol g^–1^), and a carboxylic OH group (0.21 mmol g^–1^). The phenolic OH group was dominated by C_5_-substituted (1.45 mmol g^–1^) units, followed by guaiacyl (0.81 mmol g^–1^) units. The C_5_-substituted unit includes a syringyl unit (0.78 mmol g^–1^) and a characteristic C–C bond of β–5′, 4–O–5′, and 5–5’linkage units. The majority of syringyl units observed in PRW-lignin correlated well with its plant taxonomy as a hardwood.^40^

From the GPC results, the determined weight-averaged molecular weight (*M*_w_), number-averaged molecular weight (*M*_n_), and polydispersity index (PDI = *M*_w_/*M*_n_) of PRW-lignin were 2.33 × 10^3^ Da, 1.62 × 10^3^ Da, and 1.44, respectively (Table 1).

**Table 1:** Physiochemistry and Thermal Properties of PRW-Lignin

properties	lignin content (wt %)	ash content (wt %)	*M*_w_ (Da)	*M*_n_ (Da)	PDI	*T*_g_ (°C)
PRW-lignin	98.86	0.22	2336	1619	1.443	140.3

The glass-transition temperature (*T*_g_) of PRW-lignin was determined using DSC. In testing, the temperature was first raised to 105 °C to ensure the removal of moisture in the lignin sample and cooled down. The lignin sample was then heated to 170 °C, in the second scan, for *T*_g_ determination. From the DSC thermogram, the determined *T*_g_ was 140.3 °C (Table 1).

Melt spinning of lignin is related to *T*_g_ and melt flow, which are mainly governed by its molecular composition and structure. It is recommended that *T*_g_ of lignin should be low enough to allow melting and good flow upon heating.^1^ In fiber spinning, the spinning temperature should be higher than the *T*_g_ value of lignin to allow good flow yet low enough to prevent cross-linking or thermal degradation. In this work, a spinning temperature of 165 °C was employed to prepare PRW-lignin fibers. PRW-lignin exhibited good spinnability such that the fibers could be spun continuously and collected on a winder (at a speed of 10 m/min). This was correlated with NMR results on the chemical structures of PRW-lignin that it contains a high portion of syringyl hydroxyl unit, yielding a linear molecular structure and thus good melt flow. In addition, low molecular weight (2.33 × 10^3^ Da) and narrow PDI (1.44) of PRW-lignin also contributed to its good melt flow during fiber spinning.

**Figure 2:**
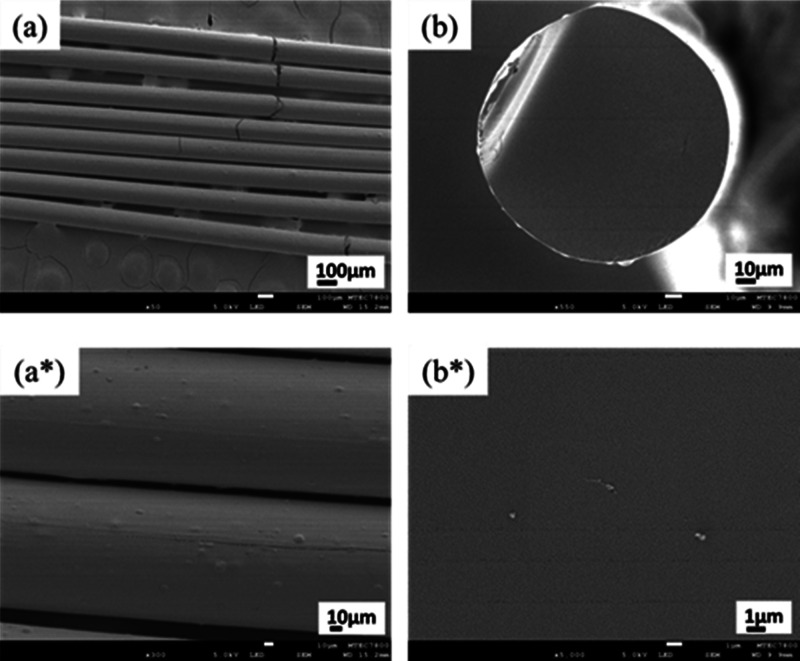
SEM micrographs of melt-spun PRW-lignin fibers, under longitudinal (a) and cross-sectional views (b) and at a higher magnification (a*, b*).

The SEM micrographs of melt-spun PRW-lignin fibers, under longitudinal (a, a*) and cross-sectional (b, b*) views, are shown in Figure 2. The surface morphologies along the fiber length and cross section were smooth. Some fractures were observed, reflecting the brittle characteristics of the amorphous lignin fiber. The average fiber diameter was 133.56 μm (Table 2).

**Table 2:** Averaged Fiber Diameters and Weight Loss of PRW-Lignin

fibers	diameter (μm)	weight loss (%)
as-spun fibers	133.56 ± 2.37	—
thermo-stabilized fibers	115.02 ± 3.28	45.44
carbonized fibers (900 °C)	84.93 ± 2.57	73.57
carbonized fibers (1000 °C)	80.56 ± 0.34	78.25
carbonized fibers (1200 °C)	75.54 ± 2.81	88.75

Thermo-stabilization is a necessary step to increase the thermal stability of lignin fibers such that it can withstand high temperatures in the carbonization process. In this study, thermo-stabilization of the PRW-lignin fibers was performed to determine the optimal heating rate. The melt-spun fibers were heated from room temperature to 250 °C, using different heating rates at 0.1, 0.2, 0.3, and 0.5 °C/min, under O_2_ flow (1 L/min).

**Figure 3:**
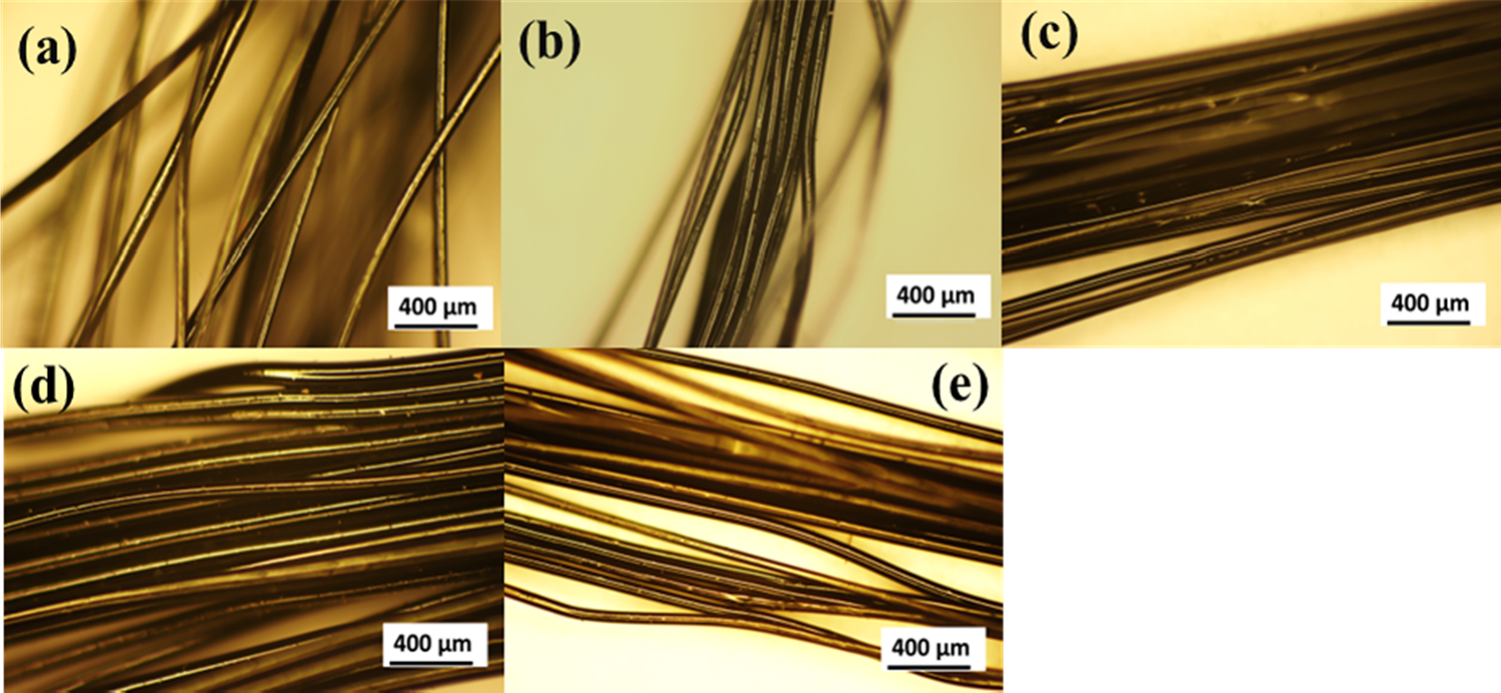
OM pictures of PRW-lignin fibers after thermo-stabilization at different heating rates: (a) 0.1 °C/min, (b) 0.2 °C/min, (c) 0.3 °C/min, (d) 0.4 °C/min, and (e) 0.5 °C/min.

From the results, it is observed that at a heating rate of 0.1 °C/min, PRW-lignin fibers exhibited thermal stability during thermo-stabilization such that no fiber fusion was observed, as shown in Figure 3a. At higher heating rates (0.2, 0.3, 0.4, 0.5 °C/min), however, fiber fusion along the surface was observed (Figure 3b–e). Fiber fusion resulted from oxidation, and cross-linking reactivity occurred during thermal stabilization. During the stabilization process, oxygen that infused into lignin fibers and oxygen atoms in lignin molecules contributed to the formation of carbonyl and carboxyl groups, yielding cross-linking between lignin molecules via anhydride and ester linkages.^12^ The heating rate employed for thermo-stabilization needs to be slow enough to maintain *T*_g_ above the stabilization temperature and prevent fiber fusion as the temperature ramps up.

**Figure 4:**
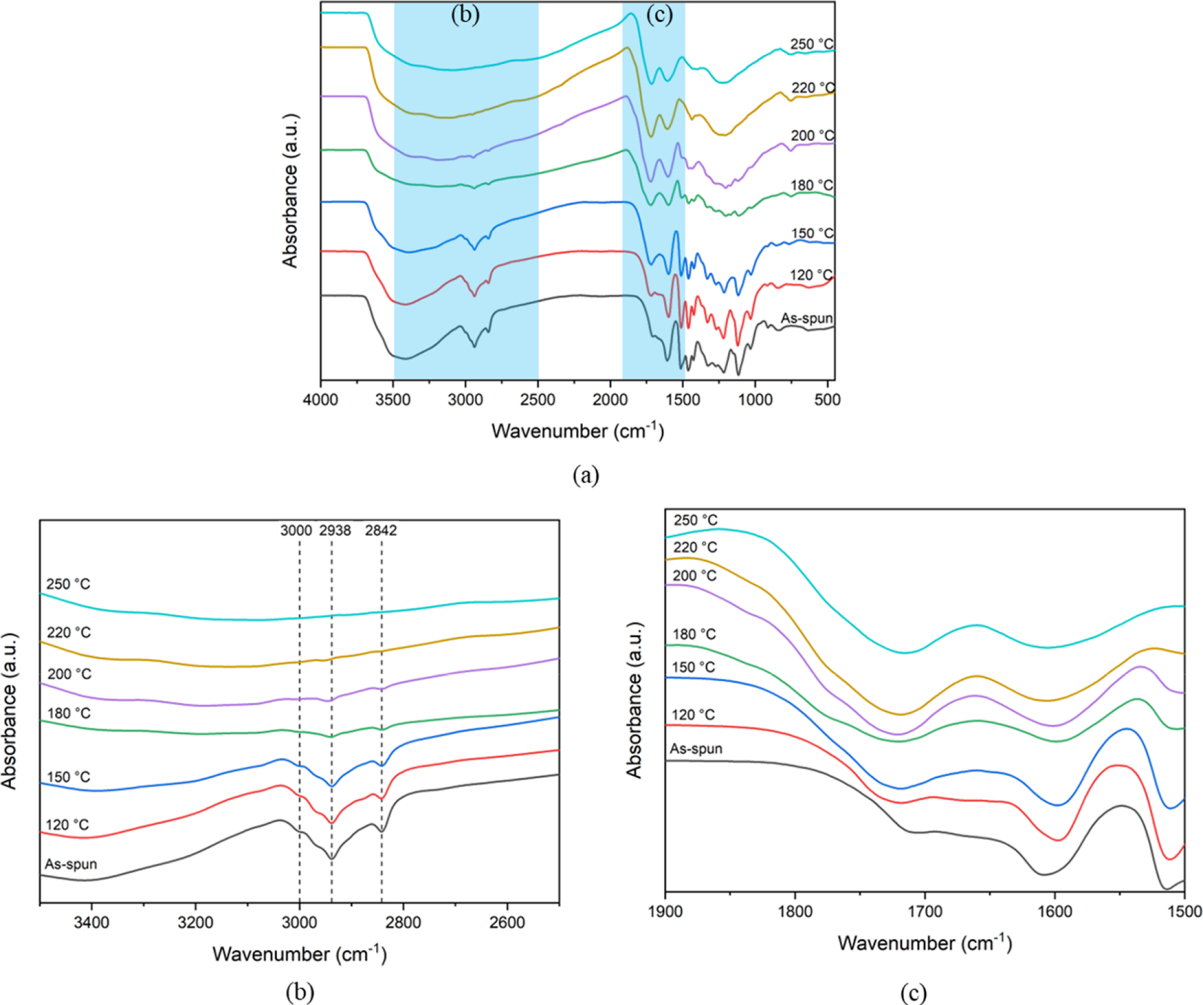
FTIR spectra of PRW-lignin fibers stabilized at different temperatures (heating rate of 0.1 °C/min): (a) whole spectra range; enlarged view (b) from 3500–2500 cm^–1^ and (c) 1900–1500 cm^–1^.

FTIR spectroscopy was conducted to investigate the structural changes during the thermo-stabilization of PRW-lignin fibers. The fibers were stabilized under oxygen atmosphere at a controlled heating rate of 0.1 °C/min from 30 to 250 °C. During heating, the fibers were sampled out when the temperature reached the designed values (120, 150, 180, 200, 220, and 250 °C). Figure 4 shows the FTIR spectra of PRW-lignin fibers stabilized at different temperatures compared to that of as-spun PRW-lignin fibers.

The functional groups detected by FTIR analysis were assigned according to the literature.^15,41−44^ The key functional groups including O–H stretching of aromatic and aliphatic hydroxyl groups (at 3450–3350 cm^–1^), aromatic C–H stretching (at approximately 3000 cm^–1^), C–H stretching in methoxy groups and methyl/methylene groups (at 2938 and 2842 cm^–1^), unconjugated C=O stretching of carbonyl groups (at 1710–1720 cm^–1^), and vibration of aromatic skeleton C–C (at 1590 cm^–1^) were followed. A broad peak of O–H stretching was detected in the as-spun PRW-lignin fibers and all stabilized fibers, while the fibers stabilized at a temperature above 150 °C showed the reduction of the hydroxyl group. This was described as due to phenol formation from the phenoxy radical generated by ether homolysis at the β–O–4′ position.^43,45^ The relative intensity of C–H stretching (at 2938 and 2842 cm^–1^) and aromatic C–H stretching (at approximately 3000 cm^–1^) decreased from 1.49 to 0.99 when the stabilization temperature increased from 120 to 200 °C. This was attributed to the increase in the aromaticity. The intensity of C–H stretching (at 2938 and 2842 cm^–1^) and aromatic C–H stretching (at approximate 3000 cm^–1^) disappeared when the stabilization temperature was raised up to 220 and 250 °C, ascribed to the oxidation reaction of alkyl groups and condensation of the aromatic ring.^43^ The band intensity at 1710–1720 cm^–1^, related to the unconjugated C=O stretching of carbonyl and carboxylic groups, increased with the increasing stabilization temperature. The increase in C=O observed in the stabilized PRW-lignin fibers agreed well with the FTIR data reported in the literature^46^ and it was described due to the auto-oxidation reaction in the presence of oxygen-based radicals.^42,47^ The absorption intensity ratio of C=O stretching at 1710–1720 cm^–1^ and vibration of aromatic skeleton C–C at 1590 cm^–1^ (*I*_C=O_/*I*_C–C_), representing the degree of oxidation of each stabilization temperature, was calculated. The *I*_C=O_/*I*_C–C_ ratio of the as-spun PRW-lignin fibers was 0.55, while that of the stabilized fibers (at 180 °C) increased to 1.01 and remained the same for those stabilized at temperatures beyond 200 °C. Furthermore, the bands observed in the range of 1500–700 cm^–1^ were significantly reduced and disappeared when the stabilization temperature was raised up to 220 °C, whereas the broad spectrum with the band center at 1202 cm^–1^ remained the same. This indicated that the condensation of the aromatic ring occurred during thermal stabilization.^43^

**Figure 5:**
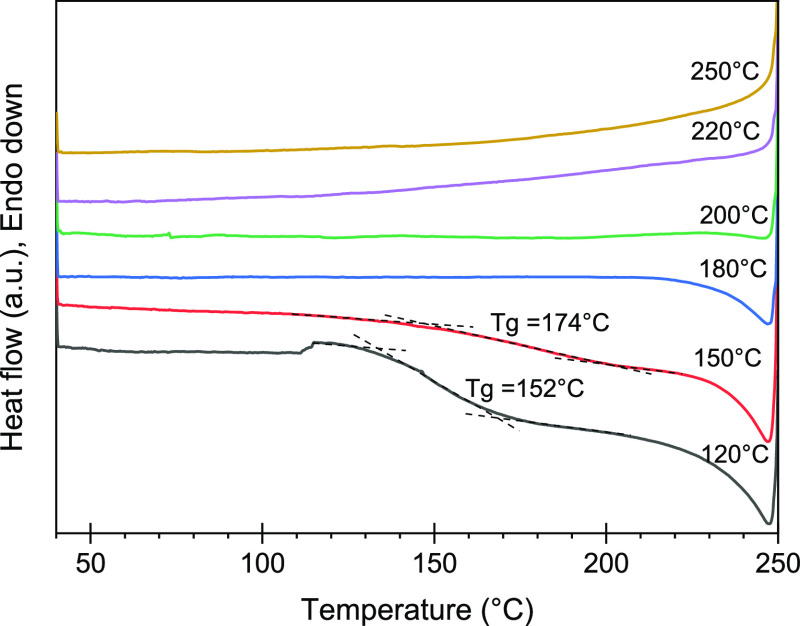
DSC thermograms of PRW-lignin fibers stabilized at different temperatures.

The glass-transition temperatures (*T*_g_) of thermo-stabilized PRW-lignin fibers were measured to investigate the changes in their thermal characteristics. The as-spun PRW-lignin fibers exhibited *T*_g_ of 138 °C, which was comparable to that of the pristine PRW-lignin (140 °C). This implied that the chemical structure of PRW-lignin had not been altered during fiber melt spinning. The thermal and structural characteristics of the lignin fibers had been converted by oxidative thermo-stabilization. The cross-linked structures in thermo-stabilized lignin fibers help maintain the fiber form during the subsequent carbonization. Figure 5 shows the DSC thermograms of PRW-lignin fibers sampled at different stabilization temperatures. An increase in *T*_g_ was observed in the fibers stabilized with a higher temperature. For example, PRW-lignin fibers thermo-stabilized at 120 and 150 °C exhibited *T*_g_ of 152 and 174 °C, respectively. Such higher *T*_g_ of stabilized fibers, compared to stabilization temperatures (120 and 150 °C), indicated that fiber fusion could be prevented. It is worth noting that *T*_g_ could not be detected when the stabilization temperature was raised up to 180 °C. This was likely due to an increase in aromaticity in the fibers, confirmed earlier by FTIR analysis.

**Figure 6:**
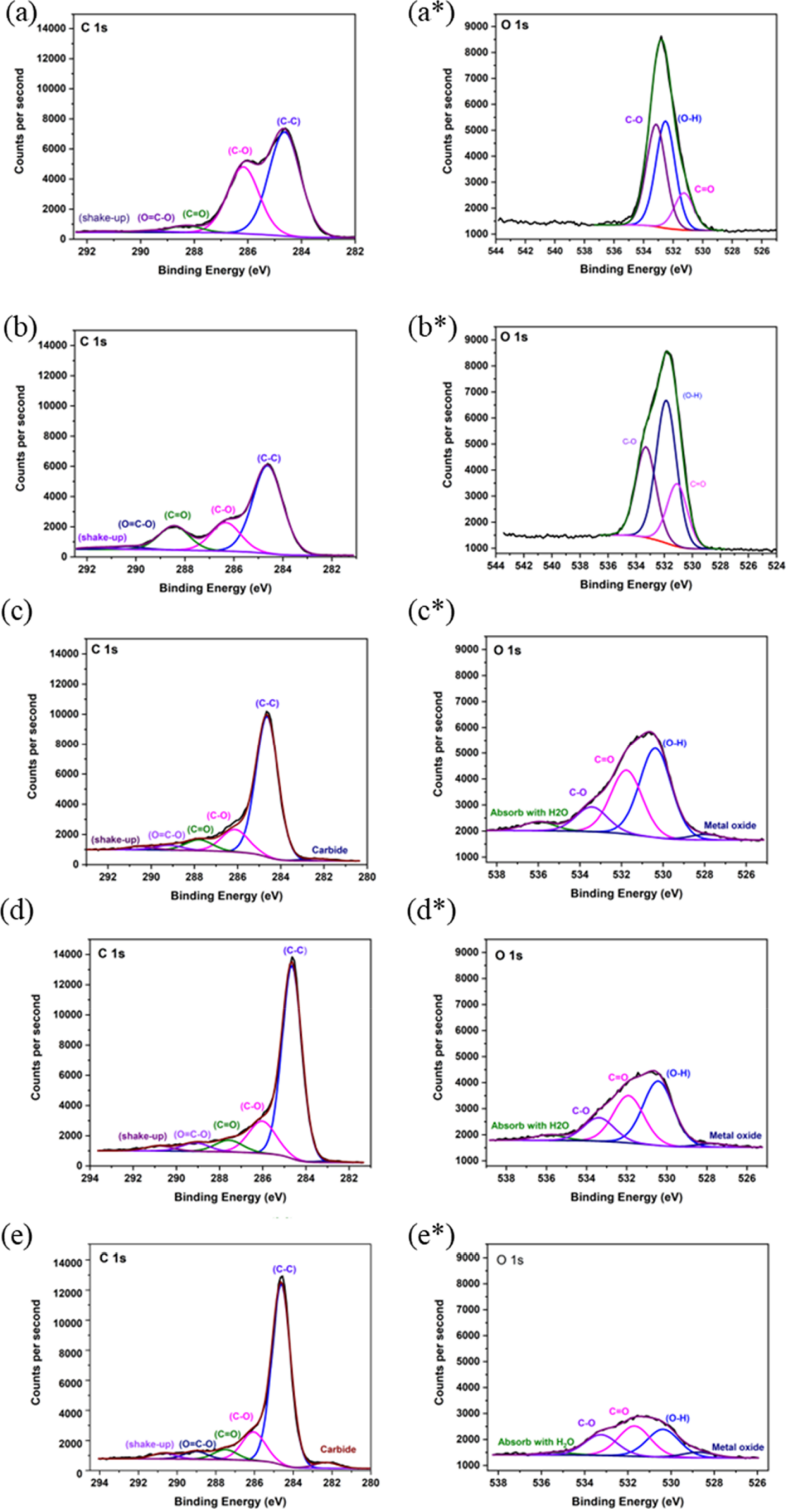
C(1s) and O(1s) XPS spectra: (a) as-spun PRW-lignin fibers, (b) stabilized PRW-lignin fibers, and PRW-lignin fibers carbonized at (c) 900 °C, (d) 1000 °C, and (e) 1200 °C.

Evidence of changes in lignin chemical structures after stabilization was detected in both C(1s) and O(1s) XPS spectra. Deconvolution of high-resolution XPS spectra of C(1s) and O(1s) was proceeded to determine the bonding types between carbon and oxygen. Figure 6a,b shows the deconvoluted components of as-spun PRW-lignin fibers and stabilized PRW-lignin fibers, respectively. After stabilization, broad C(1s) spectra observed in stabilized fibers were related to a significant decrease of C–O and increase of C=O. A similar trend was found in O(1s), as shown in Figure 6b,b*. The reduction of C–O concentration under oxidative conditions involves the cleavage of ether interunit linkages by the homolysis reaction.^42,45,48,49^ The presence of the C=O band corresponds to the formation of the carbonyl group.^42,43,49^ These results were consistent with the FTIR analysis.

**Figure 7:**
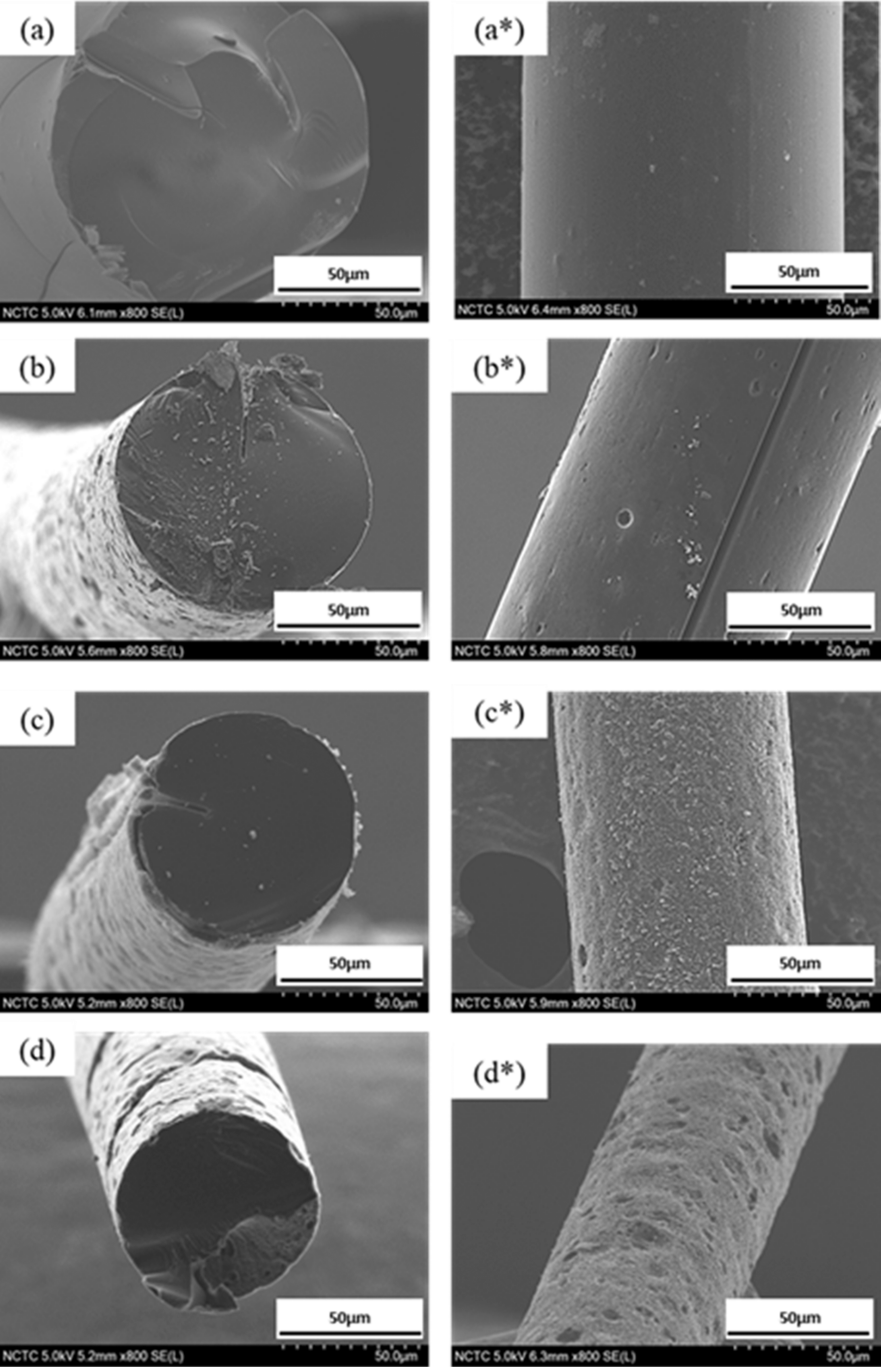
Cross-sectional and longitudinal views of (a, a*) stabilized PRW-lignin fibers and the fibers carbonized at 900 °C (b, b*), 1000 °C (c, c*), and 1200 °C (d, d*).

**Figure 8:**
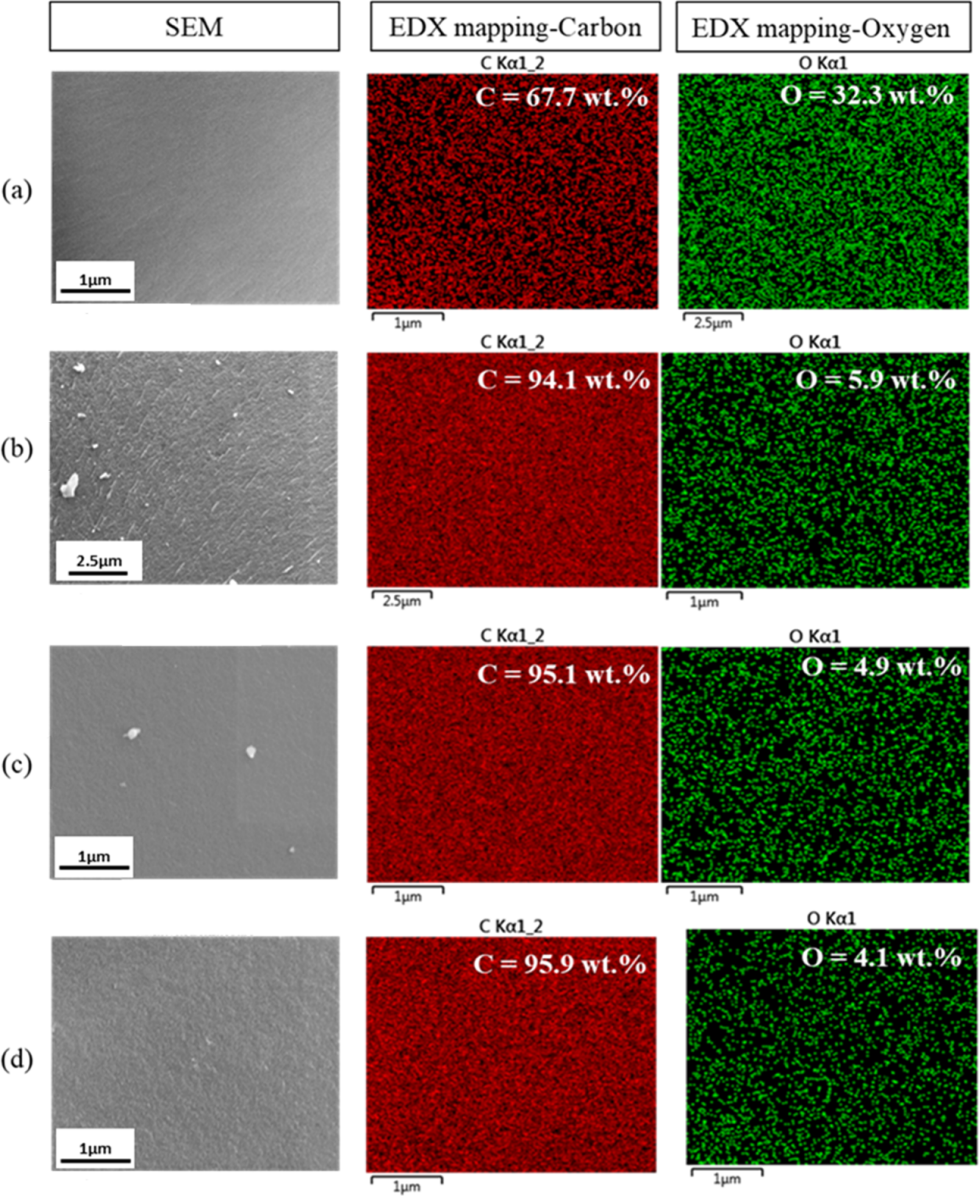
SEM images and EDX mapping images of (a) stabilized PRW-fibers and the fibers carbonized at (b) 900 °C, (c) 1000 °C, and (d) 1200 °C.

In thermo-stabilization, PRW-lignin fibers were heated from 30 to 250 °C at a heating rate of 0.1 °C/min under an O_2_ flow (1 L/min). After stabilization, PRW-lignin fibers had a reduced diameter (115.02 ± 3.28 μm) and remarkable weight loss (45.44%), compared to those of as-spun PRW-lignin fibers (133.56 ± 2.37 μm). The surface morphologies under cross-sectional and longitudinal views of the stabilized PRW-lignin fibers are displayed in Figure 7a,a*. A smooth fiber surface, similar to that of as-spun PRW-lignin fiber, was observed. The elemental composition of stabilized fibers was investigated by an EDX system equipped with a SEM instrument. Carbon and oxygen contents were the major components found in stabilized fibers, which were 67.7 and 32.3 wt %, respectively (Figure 8a).

Fiber diameters and weight losses of PRW-lignin fibers carbonized at different temperatures (900, 1000, and 1200 °C) are summarized in Table 1. After carbonization, reduced fiber diameter and increased weight loss were observed. This was explained due to the removal of noncarbon atoms and conversion into aryl carbon structure in lignin fibers upon increasing the temperature during carbonization.^50^ The fiber diameter of the PRW-lignin fibers carbonized at 900 °C was 84.93 ± 2.57 μm which became reduced to 75.54 ± 2.81 μm when a higher carbonization temperature of 1200 °C was employed.

Surface morphologies in the cross-sectional view and longitudinal view of the attained fibers carbonized at 900, 1000, and 1200 °C are shown in Figure 7b–d,b*–d*. Smooth surface was observed in the cross-sectional view, while rough and imperfected surface was observed along the fiber length in all carbonized fibers. The porous surface was remarked in the fibers carbonized at 1000 and 1200 °C, implying that the thermal degradation of disordered carbon could occur at a higher carbonization temperature.^10,51,52^ Elemental compositions of the fibers were investigated by EDX, revealing a significant increase in carbon content after carbonization (Figure 8). The carbon content in stabilized PRW-lignin fibers was determined to be 67.7 wt %, whereas those in the fibers carbonized at 900 and 1200 °C were 94.1 and 95.9 wt %, respectively. The analysis of the elemental composition of carbonized fibers was conducted by XPS. The wide-scan spectra of carbonized fibers exhibited two peaks at approximately 285.0 and 532.0 eV, representing carbon and oxygen, respectively. When the carbonization temperature was increased from 900 to 1200 °C, an increase in carbon content from 72 to 87 wt % and a decrease in oxygen content from 28 to 13 wt % were observed. The tendency of increase in carbon content and decrease in oxygen content observed in this study agreed with those reported in the literature.^53−57^

The high-resolution XPS spectra of the C(1s) and O(1s) peaks were deconvoluted to determine the chemical state of carbon and oxygen on the fiber surface. Figure 6c–e shows the deconvoluted components of PRW-lignin fibers carbonized at 900, 1000, and 1200 °C, respectively. A significant decrease in C=O was observed in carbonized fibers, compared to that in stabilized fibers (Figure 6b–d). Moreover, increasing carbonization temperature resulted in a decrease in C=O, as observed in both the C(1s) (Figure 6c,d) and O(1s) (Figure 6c*–d*) regions. On the other hand, an increase in C–C composition was observed when the carbonization temperature increased from 900 to 1000 °C, indicating an increase in aryl and condensed aryl groups (Figure 6c,d). However, the C–C composition slightly dropped when the carbonization temperature was further increased to 1200 °C. This indicated that the decomposition of carbon could occur at such a high temperature. The evidence for carbon decomposition was given by the formation of C–O when the temperature reached 1200 °C, as shown in Figure 6d*. The O(1s) spectra shifted to a higher bonding energy, indicating the reduction of O–H, and the structure favors C–O formation. The XPS results reported in this study agreed well with the ^13^C NMR data reported by Foston et al.^58^

**Figure 9:**
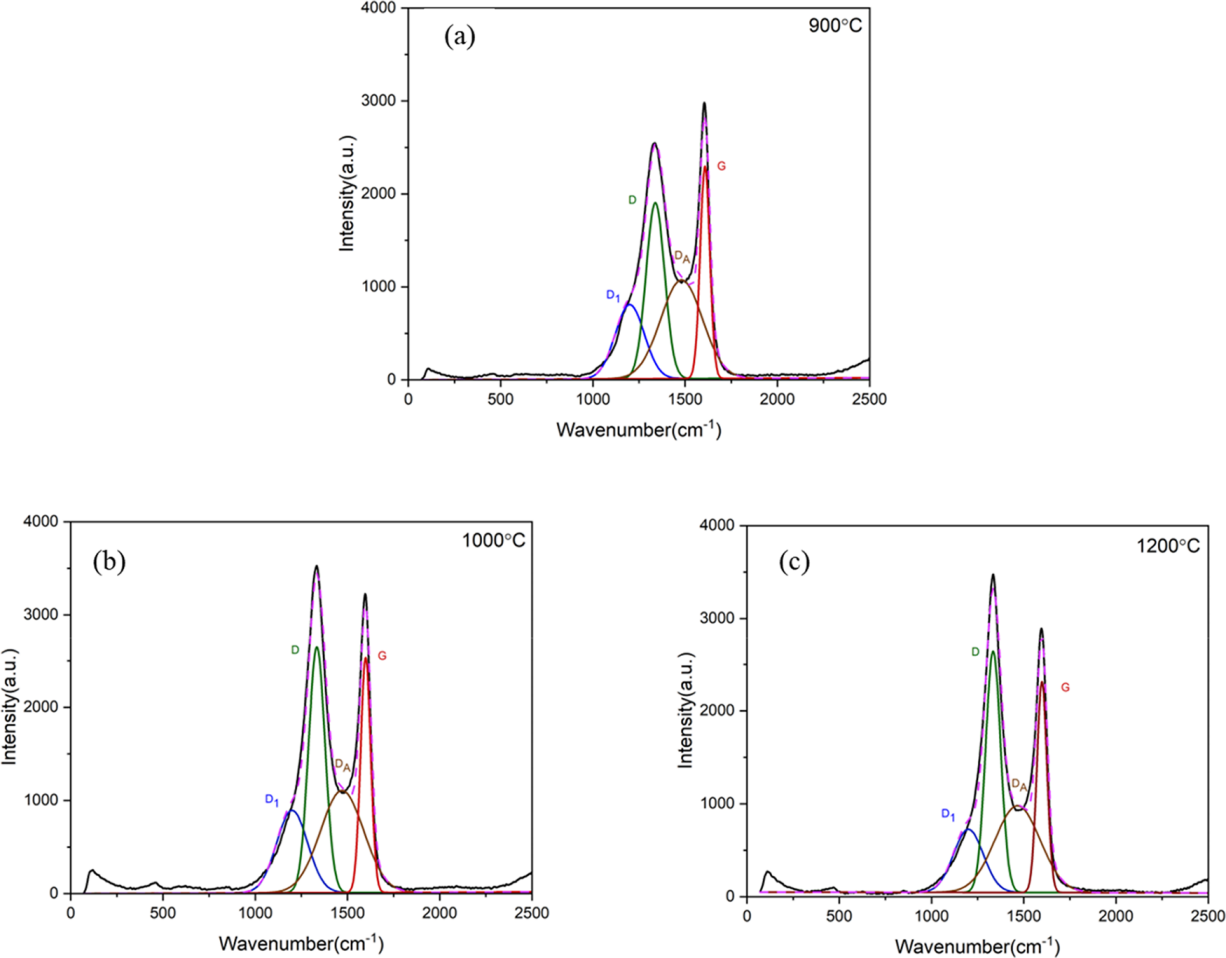
Raman spectra of the surface of the PRW-lignin fibers carbonized at (a) 900 °C, (b) 1000 °C, and (c) 1200 °C.

Raman spectroscopy has been widely used to study the structures of carbon materials.^59−61^ The Raman spectra of the PRW-lignin fibers carbonized at 900, 1000, and 1200 °C are shown in Figure 9. Carbonized fibers exhibited two characteristic bands, with the band peaks at ≅1358 cm^–1^ (D-band) and at ≅1589 cm^–1^ (G-band), overlapping each other. D-band represents the valence state of sp^3^ hybridization, relating to disordered carbon, while G-band represents the valence state of sp^2^ hybridization, relating to ordered carbon.^59,61−63^ The overlapped Raman spectra were deconvoluted to extract four subbands of D_I_ (at 1100–1200 cm^–1^), D (1340–1360 cm^–1^), D_A_ (1450–1500 cm^–1^), and G (1580–1600 cm-1).^59−61^ The extracted information including intensity, integrated area, and full width at half-maximum (FWHM) of D-band and G-band are summarized in Table 3.

**Table 3:** Intensity, Integrated Area, and FWHM of D-Band and G-Band and the *I*_D_/*I*_G_ and *A*_D_/*A*_G_ Ratios

carbonization temperature	D-band			G-band			*I*_D_/*I*_G_	*A*_D_/*A*_G_
	intensity	area	FWHM	intensity	area	FWHM		
900 °C	2566.62	455,062.51	166.56	2900.53	239,158.90	77.46	0.88	1.90
1000 °C	2867.96	359,850.24	117.87	2653.96	213,171.79	75.46	1.08	1.69
1200 °C	2668.72	278,455.40	98.02	2274.65	160,011.07	66.08	1.17	1.74

The ratio of D-band intensity to that of G-band (*I*_D_/*I*_G_) represents the degree of carbon structure ordering. A lower *I*_D_/*I*_G_ value indicates good ordering, whereas a higher *I*_D_/*I*_G_ value refers to poor ordering. *I*_D_/*I*_G_ of the fibers carbonized at 900 °C was 0.85 which increased to 1.09 and 1.15 when the carbonization temperature increased to 1000 and 1200 °C, respectively. Similar observation was reported in other works on carbonized lignin precursors.^33,53,66^ For example, softwood kraft lignin (SKL): softwood kraft pulp (KP)-derived carbon fibers exhibited an increase in *I*_D_/*I*_G_ from 0.7 to 1.0 when the carbonization temperature was increased from 600 to 1000 °C. Similarly, the carbonized fiber of neat solvent-fractionated softwood kraft lignin exhibited an increased *I*_D_/*I*_G_ from 0.82 to 0.91 and 1.04 when the carbonization temperature was increased from 1000 to 1200 and 1500 °C, respectively.^67,68^ This suggested the formation of a disordered structure in carbonized lignin fibers, which was ascribed to the irregular and complex structures of lignin that inhibited the rearrangement of the ordered graphitic structures.^53,66,69−71^

Thermal decomposition of carbon was also observed by the reduction in crystallite size.^67,68^ The I_D_/I_G_ ratio was used to calculate the crystallite size using eq 1:^51^
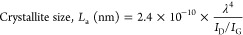
1where λ is the output wavelength of the laser in nanometers (532 nm). The calculated *L*_a_ values of the attained fibers carbonized at 900, 1000, and 1200 °C were 22.6, 17.6, and 16.6 nm, respectively. The reduction in crystallite size as well as an increase in disordered carbon with sp^3^ hybridization indicated the thermal decomposition of carbon. The FWHM of both D-band and G-band decreased with the increasing carbonization temperature, suggesting that both disordered carbon and ordered carbon were uniform.

To further explore its utilization as a carbon fiber precursor, the mechanical properties of the carbonized PRW-lignin fibers were observed. From the results, the tensile strength and Young’s modulus of thermo-stabilized precursor fibers were observed to be 22.2 ± 4.8 and 20.4 ± 3.4 MPa, respectively. After carbonization, the fibers carbonized at 900, 1000, and 1200 °C exhibited an increased tensile strength of 87.4 ± 3.6, 99.8 ± 32.3, and 163.0 ± 42.1 MPa and Young’s modulus of 144.8 ± 63.7, 157.8 ± 54.8, and 275.1 ± 43.4 MPa, respectively. These were relatively low, compared to those reported by other related works, where tensile strengths were in the range of 355–510 MPa.^14,72^ The lower tensile strength of the carbonized PRW-lignin fiber was suspected due to, first, the brittle nature of lignin that obstructed fiber stretching during spinning, and second, the equipment’s limitation where further fiber stretching during thermo-stabilization and carbonization could not be conducted. In our opinion, addition of heating equipment next to the spinneret may help annealing the extruded lignin, and thus further extension of the fibers during spinning could be achieved. This will be undertaken in our future work, which will focus on improving the tensile properties of the developed PRW-lignin carbon fibers via spinning finer fibers as well as the fiber conversion process.

## Conclusions

In this work, fully biomass carbon fiber was prepared from PRW-lignin. The PRW-lignin fibers were melt-spun continuously at 165 °C, followed by thermo-stabilization (250 °C) and carbonization (900, 1000, and 1200 °C). Thermo-stabilized fibers with no fusion were obtained at a heating rate of 0.1 °C/min, whereas at higher heating rates (0.2, 0.3, 0.4, and 0.5 °C/min), fiber fusion was observed. At a heating rate of 0.1 °C/min, structural changes in the fibers during the stabilization process were investigated by sampling out the fibers at 120, 150, 180, 200, 220, and 250 °C for FTIR analysis. Results revealed the structural changes in PRW-lignin fibers toward aromaticity during thermo-stabilization. Reduction of the hydroxy group was observed when the stabilization temperature was above 150 °C. DSC results showed that *T*_g_ of PRW-lignin fibers increased with increasing temperature during stabilization. Changes in chemical structures after stabilization were also confirmed by XPS, and the results were consistent with FTIR analysis results. From XPS, it is observed that the carbon content in carbonized fibers increased, while oxygen content decreased, with the increasing carbonization temperature. The high weight loss (88.7%) of fibers carbonized at 1200 °C was explained partly due to the thermal decomposition of disordered carbon. The tensile strength and Young’s modulus of carbonized fibers were 163.0 and 275.1 MPa, respectively.
